# Impacts of Nitrate and Nitrite on Physiology of *Shewanella oneidensis*

**DOI:** 10.1371/journal.pone.0062629

**Published:** 2013-04-23

**Authors:** Haiyan Zhang, Huihui Fu, Jixuan Wang, Linlin Sun, Yaoming Jiang, Lili Zhang, Haichun Gao

**Affiliations:** 1 Institute of Microbiology and College of Life Sciences, Zhejiang University, Hangzhou, Zhejiang, China; 2 College of Life Sciences, Tarim University, Alar, Xinjiang, China; Wageningen University, The Netherlands

## Abstract

*Shewanella oneidensis* exhibits a remarkable versatility in anaerobic respiration, which largely relies on its diverse respiratory pathways. Some of these are expressed in response to the existence of their corresponding electron acceptors (EAs) under aerobic conditions. However, little is known about respiration and the impact of non-oxygen EAs on the physiology of the microorganism when oxygen is present. Here we undertook a study to elucidate the basis for nitrate and nitrite inhibition of growth under aerobic conditions. We discovered that nitrate in the form of NaNO_3_ exerts its inhibitory effects as a precursor to nitrite at low concentrations and as an osmotic-stress provider (Na^+^) at high concentrations. In contrast, nitrite is extremely toxic, with 25 mM abolishing growth completely. We subsequently found that oxygen represses utilization of all EAs but nitrate. To order to utilize EAs with less positive redox potential, such as nitrite and fumarate, *S. oneidensis* must enter the stationary phase, when oxygen respiration becomes unfavorable. In addition, we demonstrated that during aerobic respiration the cytochrome *bd* oxidase confers *S. oneidensis* resistance to nitrite, which likely functions via nitric oxide (NO).

## Introduction

*Shewanella oneidensis* is a facultative Gram-negative anaerobe with remarkable anaerobic respiration abilities that allow the use of a diverse array of terminal electron acceptors (EA), including fumarate, nitrate, nitrite, thiosulfate, trimethylamine *N*-oxide (TMAO), dimethylsulfoxide (DMSO), Fe(III), Mn(III) and (IV), Cr(VI), and U(VI) [1]. In order to reduce these diverse EAs, the *S. oneidensis* genome encodes a large number of respiratory pathways, some of which have been elucidated over the last two decades [1]–[8].

In the case of nitrate and nitrite respiration, *S. oneidensis* has many unique properties. The genome of *S. oneidensis* possesses operons *napDAGHB* and *nrfA* for periplasmic nitrate reductase (NAP) and nitrite reductase (NRF), respectively [3]. Surprisingly, NapC and NrfH or the complex of NrfBCD, which in many bacteria are essential and dedicated electron transport components of NAP and NRF respectively, were missing [9], [10]. To complete these pathways, nitrate and nitrite terminal reductases recruit CymA, a cytoplasmic membrane electron transport protein, to play the role of both NapC and NrfH for electron transport from the menaquinone pool. One consequence of sharing CymA is that reduction of nitrite to ammonium does not commence until nitrate is thoroughly exhausted, resulting in a characteristic two-step reduction of nitrate [3].

Nitrate (as a precursor to nitrite) and nitrite, on one hand, have been used for centuries as preservative in meat products to inhibit the growth of bacterial pathogens and these antibacterial effects are attributed to NO formation [11]–[13]. *S. oneidensis* is capable of producing NO in the presence of either nitrate or nitrite under microaerobic/anaerobic conditions although the enzymatic machinery for NO synthesis is unknown [14]. NO interferes with biological processes by either interacting with protein cofactors, such as Fe-S clusters, heme, and lipoamide, or by promoting the formation of reactive nitrogen species (RNS) [11], [15], [16]. On the other hand, both nitrate and nitrite can support the growth of *S. oneidensis* as sole EA under anaerobic conditions although biomass production is rather limited [3]. Two mM nitrite facilitates growth most effectively, resulting in an OD_600_ up to 0.06, which cannot be reliably measured [2], [3]. Nitrate *per se* supports a greater biomass but it is rapidly converted to nitrite, which hampers further growth, also resulting in an extremely low maximum cell density (less than 0.1 of OD_600_) [2], [3]. As a consequence, mutants lacking any of the genes involved in nitrate/nitrite respiration or its regulation have further limited growth and cannot be readily studied.

Following the elucidation of anaerobic nitrate and nitrite respiration in *S. oneidensis* [3], we have undertaken a study of the regulatory proteins mediating these processes. In order to obtain adequate biomass for the characterization of mutants impaired in regulation of nitrate/nitrite respiration, we tested various aerobic cultivation conditions, including shake flasks (uncontrolled batch), controlled batch bioreactors, and chemostats [17]. Although reduction of nitrate to nitrite occurred in controlled cultures, reduction of nitrite to ammonium did not, and thus unsuitable for studies of nitrite respiration in *S. oneidensis*. Surprisingly, in batch cultures both reduction processes preceded without compromising biomass production. This simple cultivation method is useful for studies of the nitrate/nitrite respiration regulatory systems and of the mechanism underlying the differences in nitrite respiration. In this study, we investigated the impact of nitrate and nitrite on the physiology of *S. oneidensis* under aerobic conditions in order to understand their inhibitory mechanisms as well as the basis for oxygen repression on respiration of EAs with less positive redox potential, such as nitrite.

## Results

### Inhibitory Effect of Nitrate on Aerobic Growth of *S. oneidensis*

**Figure 1:**
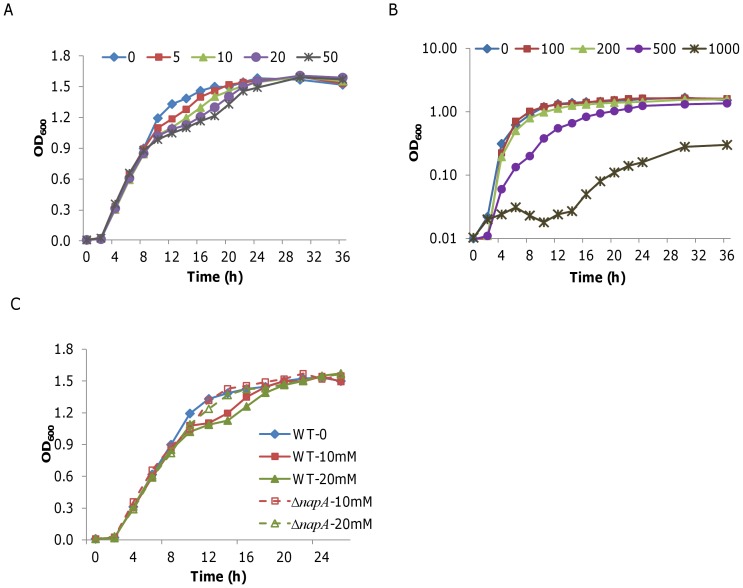
Impact of various nitrate/chloride compounds on growth of *S. oneidensis* under aerobic conditions. **A.** Growth of *S. oneidensis* in the presence of NaNO_3_ (concentrations from 5 to 50 mM). **B.** Growth of *S. oneidensis* in the presence of NaNO_3_ (concentrations from 100 to 1000 mM). **C.** Growth of *S. oneidensis* wild type and the *napA* mutant in the presence of NaNO_3_ (10 and 20 mM). All experiments were performed at least three times. Error bars, representing the standard deviation of the mean, are all less than 3% and are omitted for clarity.

Under anaerobic conditions, *S. oneidensis* is able to survive in the presence of up to 100 mM nitrate but grows to a maximum cell density (less than 0.1 of OD_600_) only with less than 4 mM [2], [3]. To evaluate the impact of nitrate on aerobic cultures of *S. oneidensis*, we monitored the bacterial growth in LB-NaCl_less_ (containing one tenth of NaCl, ∼17 mM Na^+^ to reduce the impact of Na^+^) media supplemented with NaNO_3_ at various levels. When NaNO_3_ was added at low concentrations (5–50 mM), growth of *S. oneidensis* started to deviate slightly from the standard growth curve at ∼0.8 of OD_600_ (Fig. 1A). Growth was significantly reduced when NaNO_3_ exceeded 200 mM and was barely detectable in the presence of 1 M (Fig. 1B). Notably, the maximum cell densities were indistinguishable at all test concentrations below 1 M. These data suggest that NaNO_3_ affects growth of *S. oneidensis* under aerobic conditions by two distinct mechanisms, which are dose-dependent.

To separate these two mechanisms, the same experiment was carried out with NaCl (25–1000 mM). Growth of *S. oneidensis* was not affected by NaCl at concentrations less than 100 mM but resembled that resulted from NaNO_3_ at 200 mM or above (data not shown), implicating that NaNO_3_ at the high concentrations impedes growth through sodium which functions as a salt/osmotic stress agent. Second, the experiment was repeated with KNO_3_, which provides nitrate but not sodium. As expected, KNO_3_ at the concentrations of lower than 50 mM induced the aberrant growth similar to that with 50 mM NaNO_3_ (data not shown), thus confirming that the aberrant growth is specific to NaNO_3_. To determine whether nitrate *per se* accounts for the aberrant growth of *S. oneidensis*, we repeated the experiment with a strain (Δ*napA*) lacking *napA* (encoding the large subunit of nitrate reductase), which is defective in nitrate reduction [3]. Results presented in Fig. 1C revealed that deletion of *napA* resulted in growth that was comparable to that of the wild type in the presence of nitrate, indicating that nitrate *per se* is not the cause for the aberrant growth of *S. oneidensis*.

### Reduction of Nitrate in *S. oneidensis* during Aerobiosis

**Figure 2:**
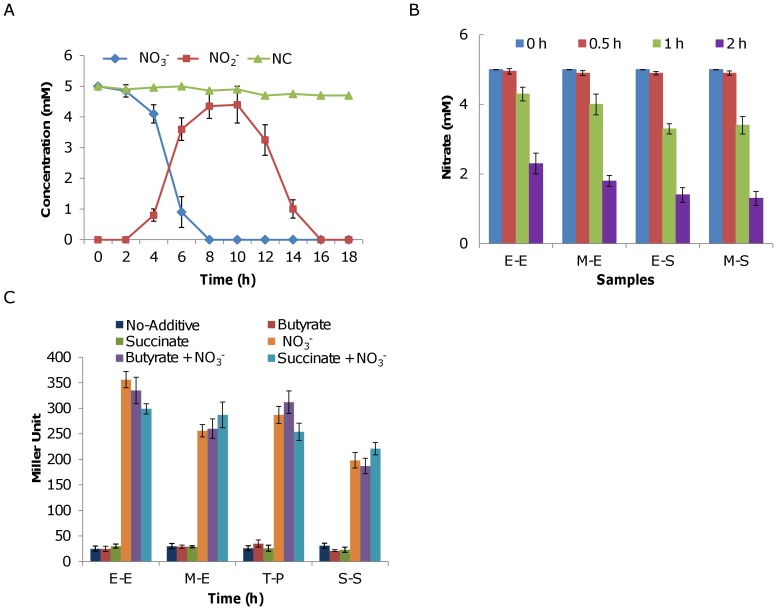
Nitrate respiration under aerobic conditions in *S. oneidensis*. **A.** Nitrate/nitrite assays of aerobic cultures. *S. oneidensis* grown in the presence of nitrate was collected at the indicated time points and the supernatants were assayed using IC. The Δ*napA* strain was used as a negative control (NC). **B.** Nitrate respiration in cultures at different growth phases in the presence of 5 mM nitrate and assayed 30 min, 1 hour and 2 hours after addition of the chemical. Early exponential phase (E-E, ∼0.2 of OD_600_), mid-exponential phase (M-E, ∼0.4), entry into the stationary phase (E-S, ∼0.8), and 4 hour after the entry into the stationary phase (M-S, ∼1.6). **C.** Impacts of reduced and oxidized carbon sources on expression of the *nap* operon using a *lacZ* reporter system. Cultures of the wild type grown to the indicated phases were supplemented with a combination of 5 mM nitrate and 10 mM sodium succinate (oxidized carbon source) and/or 10 mM sodium butyrate (reduced carbon source), collected 1 h later, and assayed for β-galactosidase activity. Experiments were performed at least three times and error bars represent the standard deviation of the mean.

As nitrate is not the agent that directly impairs growth, it is conceivable that its reduced product, nitrite, most likely caused the observed phenotype given that the *in vivo* toxicity of nitrite is well-established. To gain a better understanding of nitrate and nitrite reduction mechanism in aerobic growing cultures, we examined concentrations of nitrate and nitrite in cultures supplemented with 5 mM NaNO_3_ (Fig. 2A). Nitrite reached detectable levels 4 hours after inoculation (∼0.2 of OD_600_), was maximum approximately 8 hours after inoculation (∼1.2 of OD_600_), remained at the same level for about 3 hours, and was gone within 16 hours, indicating that *S. oneidensis* cells are able to respire on nitrite when oxygen is present. However, reduction of nitrate to ammonium in the presence of oxygen is clearly distinct from the two-step process observed under anaerobic conditions where nitrite reduction begins immediately after nitrate is completely consumed [3], [18], suggesting that certain requirements must be met for aerobic growing cells to proceed with nitrite reduction.

To test whether reduction of nitrate is dependent on the physiological status of cells, nitrate was added to cultures at various OD_600_ values: ∼0.2 (early exponential, E-E), ∼0.4 (mid-exponential, M-E), ∼0.8 (entry stationary, E-S), and ∼1.6 (∼4 h after the entry of stationary, M-S), and concentrations of nitrate and nitrite were then monitored by ion chromatography (IC). As shown in Fig. 2B, nitrate reduction occurred in all cultures, indicating that the process is independent of the growth phase of the cells.

In *Paracoccus denitrificans*, aerobic nitrate reduction by NAP is employed to dissipate excess redox energy during oxidative metabolism of reduced carbon substrates but not oxidized carbon substrates [19], [20]. On the contrary, aerobic nitrate reduction in *Rhodobacter sphaeroides* and *Thiosphaera pantotropha* is insignificant in regard of physiology and expression of *nap* is irrespective of oxygen, carbon source, and even nitrate [21], [22]. To test the role of aerobic nitrate reduction in *S. oneidensis*, experiments were repeated with the same medium supplemented with reduced carbon source butyrate or relatively oxidized carbon substrate succinate. Neither of these additives elicited a notable difference in growth or nitrate utilization (data not shown). Additionally, expression of *nap* in cells at different growth stages was examined using a P*_nap_*-*_lacZ_* reporter system [17]. Significant changes in *nap* expression were not observed when any of these carbon sources was added solely whereas the expression was elevated considerably with supplemented nitrate (Fig. 2C). These results suggest that aerobic nitrate respiration of *S. oneidensis* may not have a role in dissipation of excess redox energy.

### Inhibitory Effects of Nitrite on Growth of *S. oneidensis* Under Aerobic Conditions

**Figure 3:**
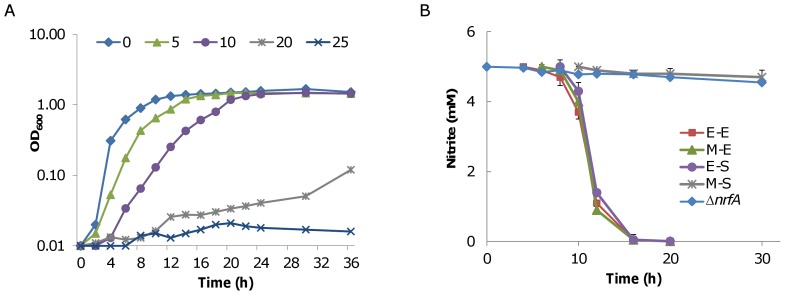
Response to nitrite during aerobic growth of *S. oneidensis*. **A.** Growth of *S. oneidensis* in the presence of NaNO_2_ (from 5 to 25 mM). Experiments were performed at least three times and error bars, representing the standard deviation of the mean, were all less than 3% and are omitted for clarity. **B.** Time-of-addition assay. 5 mM nitrite (final concentration) was added to aerobic growing cultures at E-E (∼0.2 of OD_600_), M-E (∼0.4), E-S (∼0.8), and M-S (∼0.8, 4 hour after E-S) as described in the text and the nitrite concentrations were determined at the indicated time points. Experiments were performed at least three times and error bars represent the standard deviation of the mean.

We then investigated the influence of nitrite on growth with NaNO_2_ as a nitrite provider under aerobic conditions. Not surprisingly, NaNO_2_ had a strong inhibitory effect on the growth of *S. oneidensis*. At 5 mM, there was an apparently extended lag time and a lowered growth rate whereas 25 mM completely prevented cells from growing (Fig. 3A). Since the concentrations of sodium used in the experiment is too low to elicit an osmotic effect, it is reasonable to conclude that the observed inhibitory effect results from nitrite. Meanwhile, it is worth noting that the maximum cell densities of these cultures were virtually the same if growth occurred (in the case of 20 mM, 72 h was needed to reach the maximum level).

To validate the reduction of nitrite under aerobic conditions, we repeated the experiments with an *nrfA* in-frame deletion strain (Δ*nrfA*) [3]. As expected, this strain was unable to carry out nitrite reduction, indicating that the same NRF system responsible for respiration of nitrite under anaerobic conditions also catalyzes nitrite reduction under aerobic conditions (Fig. 3B). We then performed a time-of-addition assay to examine whether cells at different growth stages were responsive to nitrite in a similar pattern. Cultures at E-E (∼0.2, OD_600_), M-E (∼0.4), E-S (∼0.8), and M-S (∼1.6) were supplemented with 5 mM nitrite (final concentration), allowed to grow under identical conditions, and the concentrations of remaining nitrite were monitored (Fig. 3B). Interestingly, it was found that i) potential for nitrite respiration was limited in cultures prior to the stationary phase, and ii) respiration of nitrite did not occur until cells had entered the stationary phase, confirming that nitrite respiration correlates with the physiological status of the cells.

### NrfA is not Sufficient for the Delay of Nitrite Reduction

**Figure 4:**
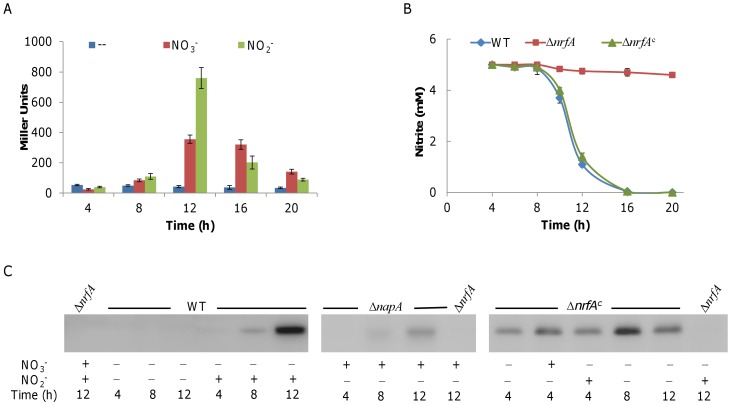
Expression of NrfA during aerobic growth of *S. oneidensis*. **A.** A *lacZ*-based reporter analysis of the *nrfA* promoter. Time of expression of *nrfA* in the Δ*napA* strain (used to block nitrate reduction) cultured aerobically with or without nitrate or nitrite. **B.** Nitrite respiration in aerobic growing cultures. Samples from cultures grown in the presence 5 mM nitrite were taken at the indicated time points and assayed for remaining nitrite. In both **B** and **C**, Δ*nrfA* ^c^ represents the *nrfA* mutant strain containing pHG102-*nrfA*, which allows constitutive expression of *nrfA*. **C.** Western blot analysis of NrfA in various samples. Cells grown for 4 (M-E), 8 (E-S), and 12 h (M-S) were collected and assayed. The Δ*nrfA* strain, grown in the presence of nitrate, nitrite, or both, was used as a negative control. To show induction of the *nrfA* gene by nitrate, the wild type (WT) was replaced by the Δ*napA* strain in order to prevent interference from conversion to nitrite. Experiments were performed at least three times. In **A** and **B**, error bars represent the standard deviation of the mean.

Discovery of an unexpected delay in nitrite reduction under aerobic conditions raised questions about the regulatory mechanisms controlling the process. To address this issue, we first examined the transcriptional level of *nrfA* at various growth stages using a P*_nrfA_*-*_lacZ_* reporter system [17]. Both nitrate (the Δ*napA* strain was used to prevent nitrate reduction) and nitrite were able to induce expression of *nrfA* (Fig. 4A). In both cases the wild-type strain expressed *nrfA* slightly before mid-stationary phase but robustly afterwards. Independent qRT-PCR analysis validated this observation (Fig. S1A).

The absence of NrfA prior to the stationary phase appears to be the cause for the delayed nitrite reduction. If so, robust expression of *nrfA* at the early growth stages should permit nitrite reduction. To test this, we transcriptionally fused *nrfA* to the *arcA* promoter which is constitutively active [18], [23] and introduced the construct into the Δ*nrfA* strain. Surprisingly, the Δ*nrfA* strain carrying the P*_arcA_*–controlled *nrfA* failed to consume nitrite before the stationary phase (Fig. 4B). As this P*_arcA_*–controlled *nrfA* complemented the Δ*nrfA* strain in its ability to respire on nitrite after the stationary phase, the expressed NrfA is therefore functional. To verify expression levels of NrfA in cells at various growing stages, we raised an antibody against the enzyme and used in cells at various growth stages. Consistent with the levels of the *nrfA* transcript, NrfA was present at an extremely low level in E-E cultures, increased significantly in cells entering the stationary phase, and maximized in M-S cultures (Fig. 4C). Apparently, the production of NrfA was inducible by either nitrate or nitrite, with the latter more efficient. More importantly, the Western blotting analysis confirmed that *nrfA* driven by P*_arcA_* was indeed expressed constitutively, independent of not only nitrate/nitrite but also of the growth phases. In total, these data indicate that NrfA *per se* is not accountable for the delayed reduction of nitrite.

### Respiratory Status is Likely Responsible for the Delayed Nitrite Reduction in *S. oneidensis*

**Figure 5:**
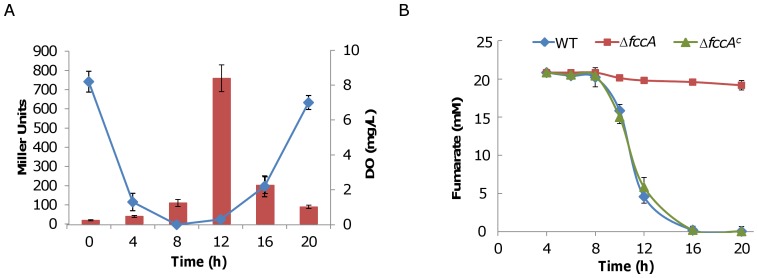
Characteristics of aerobic cultures of *S. oneidensis* **A.** Dissolved oxygen (line with diamond markers) in cultures used in Figure 4A. Expression of the *nrfA* gene (Bar) was included to show the relation of these parameters. **B.** Assay for remaining fumarate in aerobic growing cultures in the absence or presence of 20 mM. Δ*fccA^c^* represents the *fccA* mutant strain containing pHG101-*fccA*. Experiments were performed at least three times and error bars represent the standard deviation of the mean.

As the initiation of nitrite respiration is dependent on the growth phase, we speculated that the physiological status of *S. oneidensis* cells with respect to respiration may be critical to the phenotype. To test this, we measured the dissolved oxygen concentrations of cultures in the presence of nitrite. Consistent with previous findings [23], levels of dissolved oxygen in shaking cultures followed a ‘U’ curve, indicating that oxygen was saturated in the beginning, decreasing below detectable levels (exponential phase), and then increasing to saturation in the late-stationary phase (Fig. 5A). Saturation levels of oxygen in the beginning can be readily explained by the low number of cells in the culture whereas at the late stage it implies that oxygen consumption decreased substantially. Given that maximum *nrfA* expression coincides with the accumulation of oxygen in the culture, we hypothesize that *S. oneidensis* cells in the stationary phase have shifted to utilize EAs with a low reduction potential even when oxygen is available. To test this hypothesis, we cultured cells aerobically with 20 mM fumarate and assayed for fumarate reduction. As shown in Fig. 5B, with an *fccA* mutant as a negative control, cells started to respire on fumarate only after entry into the stationary phase [24]. An additional analysis of the *fccA* expression using qRT-PCR confirmed that fumarate failed to stimulate transcription of *fccA* until cells had entered the mid-stationary phase (Fig. S1B). In sum, it appears that *S. oneidensis* is unable to overcome oxygen repression in order to respire on EAs with the relatively low positive standard redox potential.

### Cytochrome *bd* Oxidase Confers *S. oneidensis* Resistance to Nitrite and NO

The antibacterial effects of nitrite are generally attributed to NO, which is reportedly produced endogenously from nitrite in *S. oneidensis* as in many prokaryotes [11], [13]–[16], [25]. A common strategy adopted by many species to overcome NO toxicity is to express multiple oxidases, one of which is less sensitive to NO [11], [26]–[28]. The genome of *S. oneidensis* encodes two cytochrome *c* terminal oxidases: SO4606-4609 (*caa*_3_-HCO) and SO2364-2361 (CcoN-O-Q-P, *cbb_3_*-HCO), and a quinol oxidase SO3286-3285 (CydA-B, *bd*-type) [6], [29]. As *SO4608* has been annotated as *coxG*, we named *SO4606*, *SO4607*, and *SO4609 coxB*, *coxA*, and *coxC*, respectively based on sequence similarities to characterized analogues. In *S. oneidensis*, *cbb_3_*-HCO dominates respiration of oxygen whereas *caa*_3_-HCO appears to be irrelevant [24], [29]. The *bd*-type oxidase, inferior to *cbb_3_*-HCO in oxygen respiration, is critical in the bacterial resistance to nitrite [30].

**Figure 6:**
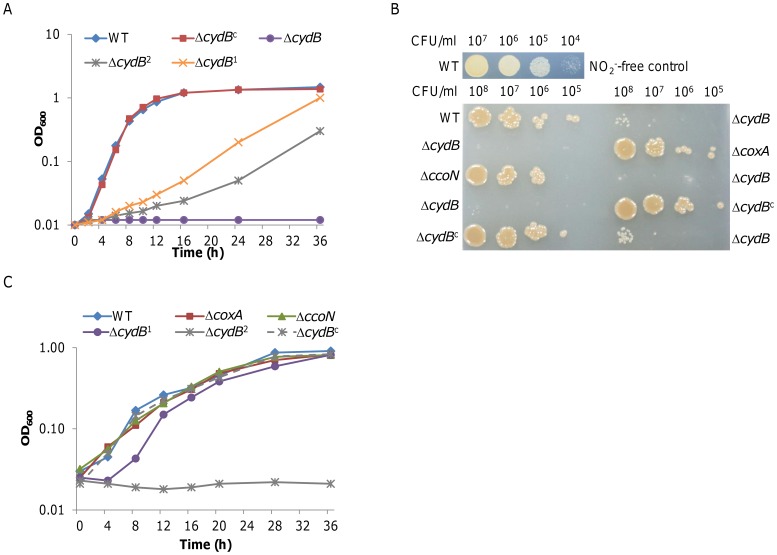
Characteristics of *S. oneidensis* strains devoid of one of the oxidases. **A.** Growth in the presence of 5 mM nitrite under aerobic conditions. Results with Δ*coxA* and Δ*ccoN*, which were identical to the wild type, are not shown. Δ*cydB* ^1^ and Δ*cydB* ^2^ represent that the Δ*cydB* strain grew in the presence of 1 and 2 mM nitrite, respectively. In all panels, Δ*cydB* ^c^ represents the *cydB* mutant strain containing pHG102-*cydB*. **B.** Sensitivity to nitrite of strains lacking one of the oxidases. Ten µl of each sample (from 10^5^ to 10^8^ CFU/ml) was spotted onto LB plates containing 5 mM nitrite. The photo was taken after 24 h incubation. The assay was repeated at least three times with similar results. WT cells spotted on the LB plates lacking nitrite were used as the reference for colony morphology. **C.** Growth of strains devoid of one of oxidases in air-tight tubes (microaerobic conditions as described in the text) in the presence of 750 µM NONOate. Δ*cydB* ^1^ and Δ*cydB* ^2^ represent that the Δ*cydB* strain grown in the presence of 750 and 1500 µM NONOate, respectively. In both **A**, and **C**, error bars, representing the standard deviation of the mean, are all less than 5% and are omitted for clarity.

To assess the possible roles that these oxidases play in nitrite/NO resistance, in-frame deletions of *coxA* (encoding the essential subunit of *caa*_3_-HCO), *ccoN* (encoding the essential subunit of *cbb*_3_-HCO), and *cydB* (encoding the essential subunit of the *bd*-type oxidase) were characterized [29], [30]. The Δ*cydB* strain displayed significantly impaired growth when challenged by nitrite at 1 mM and failed to grow with 5 mM (Fig. 6A). The observed hypersensitivity of this strain can be restored to the wild type level by expressing *cydB in trans*. In contrast, strains lacking either *coxA* or *ccoN* behaved as the wild type in the presence of 5 mM nitrite (data not shown). These observations were supported by results from the nitrite susceptibility assay (Fig. 6B). The *cydB* mutant displayed a hypersensitivity to nitrite whereas other mutants were similar in resistance to the chemical in comparison with the wild type. Notably, nitrite showed a profound impact on the physiology of *S. oneidensis*, resulting in fuzzy-looking colonies.

We then tested the effects of NO on growth of these *S. oneidensis* strains. NO was introduced to 2 ml medium in 15-ml rubber-stopped Hungate tubes by adding DETA NONOate (t_1/2,_ 20 h at 37°C and 56 h at 25°C), which releases NO slowly so there is a relatively steady NO concentration [14], [31]. Under these conditions, oxygen in the headspace enables cells to grow, allowing us to measure the inhibitory effects of NO. DETA NONOate at 1500 µM prohibited growth of the Δ*cydB* strain but not others (Fig. 6C). At 750 µM, DETA NONOate extended the lag time of the *cydB* mutant but showed no effect on growth of other mutants as well as the wild type. These data, collectively, indicate that i) nitrite, likely via NO, inhibits growth by interacting with terminal oxidases and ii) the cytochrome *bd* oxidase plays a crucial role in overcoming nitrite inhibition.

## Discussion

*S. oneidensis* is well known for its respiratory versatility, capable of respiring on oxygen or a wide range of organic and inorganic substrates under anaerobic conditions. Interestingly, there is synthesis of some anaerobic respiratory systems in the presence of oxygen, although to much lesser extent than in the absence of oxygen [32]. This implies that multiple EAs may be utilized simultaneously. Indeed, our results demonstrate that both nitrate and nitrite can be respired in agitated aerobic batch cultures despite the delay in nitrite respiration. Under conditions where the inhibitory effect of nitrite is minimal, it is possible to obtain sufficient biomass for biochemical and genetic analyses and for analyzing the degree of defectiveness of various mutants. With batch cultures under aerobic conditions, we succeeded in characterizing systems that regulate nitrate/nitrite respiration [17].

In *E. coli*, regulation of respiratory enzyme synthesis is under hierarchical control, following the standard redox potential of the electron acceptor couples (e.g. O_2_> NO_3_^–^>NO_2_^–^>fumarate) [33]. As a consequence, EAs with the most positive standard redox potential are preferentially used, a dogma that could explains why nitrate respiration is able to escape oxygen repression in *S. oneidensis*. In terms of physiological significance, aerobic nitrate reduction in *S. oneidensis*, as in *R. sphaeroides* and *T. pantotropha*, appears to play no role in dissipation of excess redox energy as observed in *P. pantotrophus* [19]–[22]. In contrast, other respiratory oxidants such as nitrite, and a group of S and N oxides exemplified by TMAO and fumarate, which are less preferentially consumed [34], are able to induce expression of their corresponding terminal reductases only after cells have entered the mid-stationary phase. In cells constitutively expressing NrfA, *S. oneidensis* cells were still unable to respire on these EAs, most likely due to the fact that oxygen outcompetes other EAs for electrons in actively growing cells. Such oxygen repression was relieved in cells that were relatively inactive with oxygen. Consequently, in these cells oxygen is no longer preferred and other EAs with less positive redox potential are utilized to drive ATP generation.

In regard to the inhibitory effects of nitrate and nitrite on *S. oneidensis*, these are substantial differences. Nitrate *per se* apparently has little impact on cells and is converted to nitrite under aerobic conditions, which is very toxic. The antimicrobial activity of nitrite is likely attributed to NO as *S. oneidensis* is able to produce NO endogenously in the presence of either nitrate or nitrite and contains a complex NO signaling pathway [14], [31]. In prokaryotes, in addition to NO synthases which are involved in the oxidation of L-arginine to NO, copper- or heme-containing cytochrome *cd*_1_ nitrite reductases and nitrate reductases (NarGHI) can also catalyze NO formation from nitrite [35]–[37]. Intriguingly, analogues to any of these enzymes have not been identified in the genome [2], [3], [14]. The enzymatic source of NO in *S. oneidensis* is not known and thus merits investigation.

NO elicits its pleiotropic effects on bacterial physiology by direct reaction with metallocofactors or through *S*-nitrosation of cysteine residues [38], [39]. To counteract NO stress, bacteria have evolved different strategies. A well understood mechanism involves a number of proteins that degrade NO such as flavogemoglobin, flavorubredoxin, and cytochrome *c*_552_ nitrite reductase, the last of which has been implicated to be in place in *S. oneidensis* [11], [31], [40]–[42]. In addition to detoxification, bacteria may synthesize multiple proteins of the same function, one of which is less sensitive to NO. A good example is terminal oxidases [11]. Some bacteria such as *Vibrio fischeri* uses alternative oxidase (AOX), which is NO-inducible and plays a specific role in NO resistance [27]. For bacteria such as *S. oneidensis* and *E. coli* lacking specific oxidases in response to NO, one of the common oxidases may assume the role of overcoming nitrosative stress [11]. The cytochrome *bd* oxidase, which is bioenergetically inferior to cytochrome *c* oxidases and plays a dispensable role in aerobic respiration, elevates tolerance to stresses elicited by both nitrite and NO [43]. It has recently been demonstrated that cytochrome *bd* is unlike HCOs in that it quickly recovers activity upon NO depletion, thereby conferring a higher tolerance to NO [11], [28], [44].

It is worth noting that the *S. oneidensis* strains devoid of cytochrome *bd* appear more sensitive to nitrite than to NO. One explanation is that the concentration of NO from DETA NONOate (no more than 0.125 µM when 200 µM NONOate is supplied) is too low to provide an inhibitory effect equal to that of 5 mM nitrite [31]. In addition, NO may be rapidly consumed by cells as the NO concentrations in control media is between 0.5 and 3 µM [31].This can explain why the cytochrome *bd* null mutant has an extended lag phase but eventually grows up, presumably after NO is reduced to a tolerable level.

## Methods

### Bacterial Strains, Plasmids, PCR Primers, and Culture Conditions

A list of all bacterial strains and plasmids used in this study is given in Table 1. Primers used for generating PCR products are available upon request. *Escherichia coli* and *S. oneidensis* strains were grown in Luria-Bertani (LB, Difco, Detroit, MI) medium at 37°C and 30°C for genetic manipulation, respectively. Where needed, antibiotics were added at the following concentrations: ampicillin at 50 µg/ml, kanamycin at 50 µg/ml, and gentamycin at 15 µg/ml.

**Table 1:** Strains and plasmids used in this study.

Strain or plasmid	Description	Reference or source
*E. coli* strain		
DH5α	Host for regular cloning	Lab stock
WM3064	Donor strain for conjugation; Δ*dapA*	W. Metcalf, UIUC
*S. oneidensis* strains		
MR-1	Wild-type	Lab stock
HG0848	*napA* deletion mutant derived from MR-1; Δ*napA*	[3]
HG0970	*fccA* deletion mutant derived from MR-1; Δ*fccA*	[24]
HG2364	*ccoN* deletion mutant derived from MR-1; Δ*ccoN*	This study
HG3980	*nrfA* deletion mutant derived from MR-1; Δ*nrfA*	[3]
HG3285	*cydB* deletion mutant derived from MR-1; Δ*cydB*	This study
HG4607	*coxA* deletion mutant derived from MR-1; Δ*coxA*	This study
Plasmids		
pDS3.0	Ap^r^, Gm^r^, derivative from suicide vector pCVD442	Lab stock
pHG101	Promoterless broad-host Km^r^ vector	[46]
pHG102	pHG101 containing the *S. oneidensis arcA* promoter	[46]
pTP327	*lacZ* reporter vector	[18]
pHG101-*nrfA*	*nrfA* is under the control of its own promoter within pHG101	This study
pHG102-*nrfA*	*nrfA* is under the control of the *arcA* promoter within pHG102	This study
pHG101-*fccA*	*fccA* is under the control of its own promoter within pHG101	This study
pHG101-*ccoN*	*ccoN* is under the control of its own promoter within pHG101	This study
pHG102-*cydB*	*cydB* is under the control of the *arcA* promoter within pHG102	This study
pTP327-P*nap*	pTP327 containing the *S. oneidensis nap* promoter	This study
pTP327-P*nrfA*	pTP327 containing the *S. oneidensis nrfA* promoter	This study

### Mutagenesis and Complementation of Mutant Strains

In-frame deletion strains were constructed using the Fusion PCR method as previously described [23]. In brief, two fragments flanking the targeted gene were amplified independently first and joined together by the second round of PCR. The resulting fusion fragment for each individual gene was introduced into plasmid pDS3.0. The resulting mutagenesis vector was transformed into *E. coli* WM3064, and then transferred into *S. oneidensis* by conjugation [45]. Integration of the mutagenesis construct into the chromosome was selected by gentamycin resistance and confirmed by PCR. Verified transconjugants were grown in LB broth in the absence of NaCl and plated on LB supplemented with 10% of sucrose. Gentamycin-sensitive and sucrose-resistant colonies were screened by PCR for deletion of the targeted gene. The deletion mutation was then verified by sequencing of the mutated region.

For complementation of genes next to their promoter, a fragment containing the targeted gene and its native promoter was generated by PCR and cloned into pHG101 [46]. For other genes, the targeted gene was amplified and inserted into the MCS of pHG102 under the control of the *arcA* promoter. Introduction of each verified complementation vector into the corresponding *S. oneidensis* mutant was done by mating with the appropriate *E. coli* WM3064 strain, and confirmed by plasmid extraction and restriction enzyme mapping.

### Physiological Characterization of the Mutant Strains

Aerobic and anaerobic growth was assayed essentially the same as described elsewhere [3]. LB-NaCl_less_ (LB containing one tenth amount of NaCl) and M1 defined medium containing 0.02% (w/v) of vitamin-free Casamino Acids were used unless otherwise noted. For anaerobic growth, 20 mM lactate served as an electron donor with one of following : NaNO_3_ (4 mM), NaNO_2_ (2 mM), and fumarate (20 mM) as an electron acceptor. To test effects of different carbon sources on aerobic nitrate/nitrite reduction, sodium succinate (10 mM final concentration) and/or sodium butyrate (10 mM final concentration) were used. Growth of *S. oneidensis* strains was determined by monitoring an increase in OD_600_ in triplicate samples. For biochemical analyses, cells were grown in 30 ml of media supplemented with NaNO_3_/NaNO_2_ (5 mM), collected by centrifugation, frozen immediately in liquid-nitrogen, and stored at −80°C for qRT-PCR, β-Galactosidase activity assay, and Western blotting and supernatants were used directly for nitrate/nitrite assays.

### β-Galactosidase Activity Assay

β-Galactosidase assays were performed using an assay kit (Beyotime, China) according to manufacturer’s instructions. Cells were washed with PBS (phosphate buffered saline) (137 mM NaCl, 2.7 mM KCl, 8.1 mM Na_2_HPO_4_ 1.76 mM KH_2_PO_4_, pH7.4), and treated with lysis buffer (0.25 M Tris/HCl, 0.5% Trion-X100, pH7.5). The resulting soluble protein was collected after centrifugation and used for enzyme assays. β-galactosidase activity was determined by monitoring color development at 420 nm using a Sunrise Microplate Reader (Tecan). The protein concentration of the cell lysates was determined using a Bradford assay with BSA as a standard (Bio-Rad). Activity was expressed in Miller units.

### Quantitative RT-PCR (qRT-PCR) Analysis

Quantitative real-time reverse transcription-PCR (qRT-PCR) analysis was carried out with an ABI7300 96-well qRT-PCR system (Applied Biosystems) as described previously [47]. The expression of each gene was determined from three replicates in a single real-time qRT-PCR experiment. The Cycle threshold (*C_T_*) values for each gene of interest were averaged and normalized against the *C_T_* value of 16s rRNA, whose abundance was consistent from early exponential to stationary phase. The relative abundance (RA) of each gene compared to that of 16s rRNA was calculated using the equation RA = 2^−Δ*CT*^.

### Immunoblotting Assay

Immunoblotting analysis was performed with rabbit polyclonal antibodies against NrfA essentially as previously described [17]. Cell pellets were washed once with PBS, and resuspended in PBS to an optical density of 0.5 at 600 nm (OD_600_) of PBS. The total protein concentration of the cell lysates was then determined by the bicinchoninic acid assay (Pierce Chemical). Samples were loaded onto SDS-10% polyacryl-amide gels and either stained with Coomassie brilliant blue or electrophoretically transferred to polyvinylidene difluoride (PVDF) according to the manufacturer’s instructions (Bio-Rad). The gels were blotted for 2 h at 60 V using a Criterion blotter (Bio-Rad). The blotting membrane was probed with anti-NrfA antibody followed by a 1∶5,000 dilution of goat anti-rabbit IgG-HRP (Horse radish peroxidase) (Roche Diagnostics) and was detected using a chemiluminescence Western blotting kit (Roche Diagnostics) in accordance with the manufacturer’s instructions. Images were visualized with the UVP Imaging System.

### Chemical Assays

Culture supernatants were subjected to Ion Chromatography (IC) for determination of nitrate and nitrite concentrations essentially as previously described [3]. The assay was performed with IonPac® AS19 with Na_2_SO_4_ as the eluent at a concentration of 100 mM with a flow rate of 0.6 ml/min in ICS-3000 (Dionex, Sunnyvale, CA). Fumarate measurements were carried out using fumarate assay kit according to the manufacturer’s instructions (Biovision, CA, USA). A standard curve was prepared each time.

### Plate Sensitivity Assay

Cells of *S. oneidensis* strains grown in LB at 30°C to an OD_600_ of ∼0.6 were adjusted to approximately 10^8^ CFU/ml with fresh LB, followed by six 10-fold serial dilutions. Ten µl of each bacterial sample (from 10^5^ to 10^8^ CFU/ml) was spotted onto the LB plates containing 5 mM nitrite. All plates were incubated at 30°C and photographs were taken about 24 hours later when colonies of the wild type were fully developed. The assay was repeated at least for three times with similar results.

## Supporting Information

Figure S1 **Expression analysis using qRT-PCR.** **A.** Transcription levels of *nrfA* in the samples used in Figure 4A. **B.** Transcription levels of *fccA* in cells grown with 20 mM fumarate to indicated time points. Experiments were performed at least three times and error bars represent the standard deviation of the mean.
(PDF)
