# Supplementary material for: Impacts of Nitrate and Nitrite on Physiology of Shewanella oneidensis
Source: PLoS One. 2013 Apr 23;8(4):e62629. doi: 10.1371/journal.pone.0062629 (PMC3633839; doi:10.1371/journal.pone.0062629)
Supplement: Figure S1 — Expression analysis using qRT-PCR. A. Transcription levels of nrfA in the samples used in Figure 4A. B. Transcription levels of fccA in cells grown with 20 mM fumarate to indicated time points. Experiments were performed at least three times and error bars represent the standard deviation of the mean. (PDF) [file pone.0062629.s001.pdf]

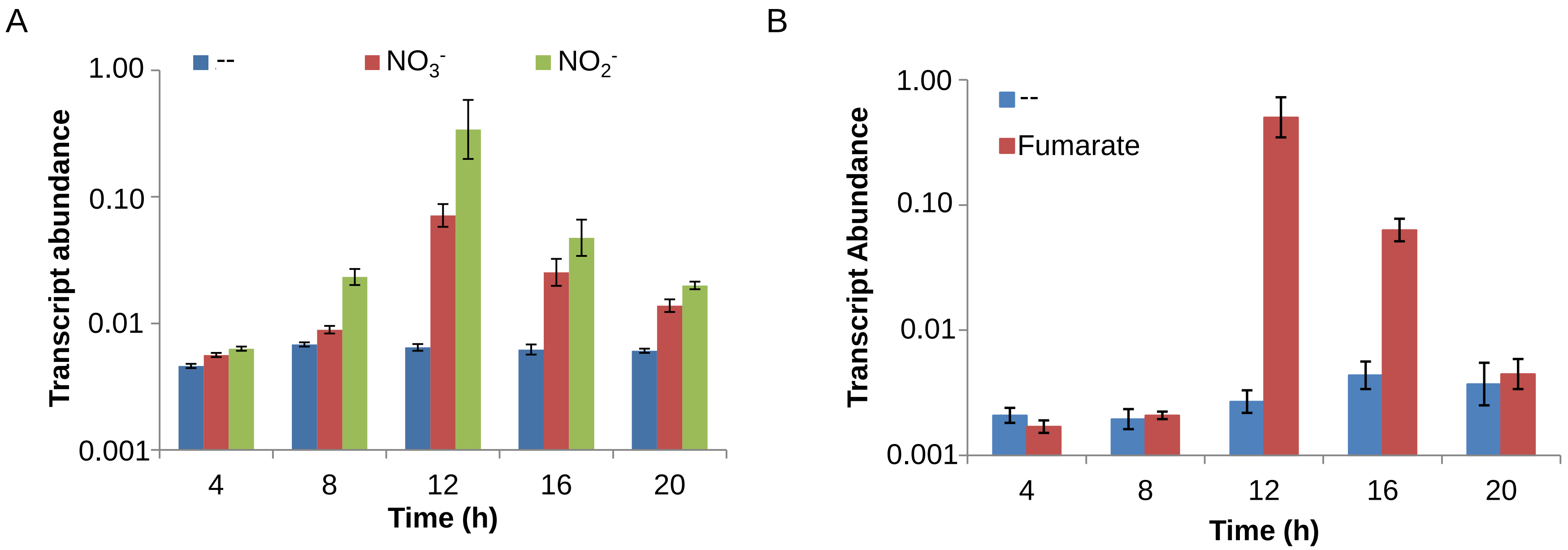

Figure S1. Expression analysis by qRT-PCR. **A.** Transcription levels of *nrfA* in the samples used in Figure 4A. **B.** Transcription levels of *fccA* in cells grown with 20 mM fumarate to indicated time points.
